# The Aqueducts and Water Supply of Ancient Rome

**DOI:** 10.1111/gwat.12958

**Published:** 2019-11-22

**Authors:** David Deming

## The Eighth Wonder of the World

One of the earliest examples of the exploitation of groundwater to sustain human civilization is the aqueduct system of ancient Rome. Although some of the aqueducts were fed by surface water, most of them were supplied by springs, usually augmented by tunneling to increase the flow of groundwater.

Diodorus Siculus enumerated the seven wonders of the ancient world in the first century BC (1814, 99). Had the accounting been taken a century or two later, surely the aqueducts of Rome would have been included. In his *Natural History*, Pliny the Elder (23 to 79 AD) touted the aqueducts of Rome as a “marvel” that was “unsurpassed” (1857, 352). “If we only take into consideration the abundant supply of water to the public, for baths, ponds, canals, household purposes, gardens, places in the suburbs, and country houses; and then reflect upon the distances that are traversed, the arches that have been constructed, the mountains that have been pierced, the valleys that have been levelled, we must of necessity admit that there is nothing to be found more worthy of our admiration throughout the whole universe” (1857, 353 to 354).

Contemplation of Roman accomplishments in hydraulic engineering compels us to agree with Pliny's assessment. And other writers were unsparing in their encomia. Greek historian Dionysius of Halicarnassus (c. 60 to 7 BC) ranked the aqueducts as one of “the three most magnificent works in Rome,” the other two being paved roads and sewers. The aqueducts were a testament to the “greatness of the Roman empire,” because of their usefulness and the expense of constructing them (Dionysius 1758, 129). The geographer, Strabo (c. 64 BC to 24 AD), noted that “so plentiful is the supply of water from the aqueducts, that rivers may be said to flow through the city and the sewers, and almost every house is furnished with water‐pipes and copious fountains” (1854, 350). Edward Gibbon (1737 to 1794), celebrated author of *The History of the Decline and Fall of the Roman Empire*, described the aqueducts of ancient Rome as “among the noblest monuments of Roman genius and power” due to “the boldness of the enterprise, the solidity of the execution, and the uses to which they were subservient” (Gibbon 1900, 70). Modern water supply systems rivalling those of ancient Rome were not constructed until the nineteenth century (Wilson 2008, 312).

The most important primary sources that inform our knowledge of the water supply of ancient Rome are inscriptions, the physical ruins of the system, and the writings of Frontinus and Vitruvius. Sextus Julius Frontinus (c. 40 to 103 AD) was appointed *curator aquarum* in 97 AD and wrote a short treatise describing his activities as supervisor of the aqueduct system (1899). Vitruvius was an engineer and architect who worked with both Julius Caesar and Augustus. Around 30 BC, he authored *De Architectura* (*On Architecture*), a work that has been more‐or‐less in continuous print for more than 2000 years.

It is tempting to equate the Roman system with modern accomplishments in hydraulic engineering and sanitation. Yet as redoubtable as their accomplishments were, the Romans nevertheless lagged far behind modern standards of health and hygienics. Life in ancient Rome was very different from that in modern times.

## Predecessors

The Romans were not the first to construct an aqueduct. Nor does it seem reasonable to regard the aqueduct itself as a novelty of any magnitude. There is nothing difficult about envisioning an artificial channel for conveying water.

Among the notable predecessors of the Roman water system was the aqueduct at Jerwan constructed by Sennacherib (reigned 704 to 681 BC), king of Assyria. Sennacherib's aqueduct was part of a regional water supply system consisting of several canals designed to supply the city of Nineveh (Fales and Fabbro 2014, 65). Sennacherib loved nature, parks, and gardens (Jacobsen and Lloyd 1935, 33). The abundant flow of water provided by the aqueduct at Jerwan and the canals facilitated the transformation of Nineveh “into a garden of almost paradisiac fertility” (Jacobsen and Lloyd 1935, 31). Around 700 BC, Sennacherib boasted “the fruits of all lands, herbs, and fruit‐bearing trees, I set out for my subjects” (Jacobsen and Lloyd 1935, 34). To solve the problem of seasonal overflow from the canals, Sennacherib went so far as to construct an artificial wetland and populate it with fauna and flora from the Babylonian marshes (Jacobsen and Lloyd 1935, 34).

The Assyrian aqueduct at Jerwan was constructed to allow canal water to pass over a ravine. It was 9 m in height, 22 m wide, and 280 m in length (Jacobsen and Lloyd 1935, 6). Water flowed through an open channel at the top of a structure about 40 cm deep fabricated from concrete and inlaid with paving stones. The Assyrian engineers obtained a remarkably constant gradient of a fall in elevation of about 12.5 m/km (Jacobsen and Lloyd 1935, 13). This is an appreciably steeper than the gradients found in Roman aqueducts, which tend to vary between 1.5 and 3.0 m/km (Hodge 1992, 218). Jacobsen and Lloyd (1935, 6) estimated that some 2 million blocks of stone were employed in construction of the aqueduct at Jerwan. A recent reassessment reduces this number to 443,520, but the “quantity of stone material theoretically required remains utterly remarkable” (Fales and Fabbro 2014, 68).

Another notable early achievement in hydraulic engineering is the qanat. A *qanat* is an underground tunnel that transports water from a well to the ground surface (Deming 2002, 136). As early as the ninth century BC “Assyrian water engineers were able to dig tunnels several kilometers long” (Wilson 2008, 293). Qanats appeared in the Middle East sometime in the early first millennium BC; the precise location and date of their origin is uncertain (Wilson 2008). At the Roman city of Timgad in North Africa, aqueducts were supplied by qanats (Hodge 1992, 22). Qanats remain in use today, commonly in arid regions (Wilson 2008, 291).

From the sixth century BC through the fourth, Athens was supplied with water by the Peisistratean, Hymettos, and Acharnian aqueducts (Chiotis and Marinos 2012, 22). Plutarch mentions that Themistocles (524 to 459 BC) fined people for “diverting the public water by pipes for their private use” (Plutarch 1887, 196 to 197). However Greek aqueducts consisted of little more than underground terracotta pipes (Hodge 1992, 25). Although the ancient Greeks made seminal contributions in philosophy, science, and mathematics (Deming 2010), the magnitude and technological refinement of their hydraulic engineering works lagged far behind Roman accomplishments. The Greeks also seemed to lack effective municipal sewers. Aristotle (384 to 322 BC) noted that in Athens one of the responsibilities of the city commissioners was to ensure that individual households did not discharge waste water into the public streets (1984, 2372).

The best known example of a Greek aqueduct, however, is not in Greece, but on the island of Samos (Deming 2010, 27 to 28). The Tunnel of Eupalinos was constructed in the sixth century BC for the purpose of bringing water from a spring into the major city on Samos (Wilson 2008, 294). According to Herodotus (c. 484 to 425 BC), the aqueduct was one of “three of the greatest works in all Greece” (1910, 239). The construction of the aqueduct involved cutting a tunnel through a hill of solid rock by excavating from both sides simultaneously. The tunnel itself did not carry water. After the adit was cut, a sloping trough was hewn into the floor. Water was then conveyed in a terracotta pipe laid in the trough. The expansive dimensions of the tunnel allowed room for human access and maintenance of the aqueduct, a feature shared with Roman aqueducts (Hodge 1992, 27 to 28).

The predecessors of the Romans in Italy, the Etruscans, had no aqueducts, but excelled at constructing drainage tunnels known as *cuniculi* (Hodge 1992, 45 to 46). The typical function of an Etruscan *cuniculi* was to remove excess water from arable land. It seems that the Romans inherited some expertise in drainage from the Etruscans, as the first major work of hydraulic engineering in ancient Rome was the great sewer, the *Cloaca Maxima*.

## The Aqueducts

Water was important in Roman culture. Vitruvius noted that water supplied “an infinite number of practical needs,” and that “all things depend upon the power of water” (1960, 226). Furthermore, “Romans relished the pleasure of water” in their baths and ornamental fountains (Rogers 2018, 83). The abundant supply of water provided by the aqueducts allowed the city of Rome itself to grow and prosper (Wilson 2008). Over a little more than 500 years, 11 aqueducts were constructed to supply ancient Rome with water (Van Deman 1934; Bruun 1991, 97 to 98). The first aqueduct was the *Aqua Appia*, erected in 312 BC by the censor Appius Claudius Caecus (c. 340 to 273 BC). During the Republican period, three more aqueducts were built: the *Anio Vetus* (272 to 269 BC), *Aqua Marcia* (144 to 140 BC), and *Aqua Tepula* (126 to 125 BC) (Bruun 2013, 298).

During the troubled first century BC, the aqueducts were largely neglected. The ascension of Augustus (63 BC to AD 14) marked a period of renewed refurbishment and construction (Forbes 1956, 670). In the days of the early Empire, three aqueducts were constructed under the supervision of Marcus Agrippa (64/62 to 12 BC): the *Aqua Julia* (33 BC), the *Aqua Virgo* (19 BC), and the *Aqua Alsietina* (2 BC). According to Frontinus, Agrippa also “rebuilt the nearly ruined aqueducts of Appia, of Anio, and of Marcia,” and “supplied the city with a large number of ornamental fountains” (Frontinus 1899, 13). The water of the *Aqua Alsietina* derived from a lake, not a spring, and was characterized by Frontinus as “unwholesome,” and unsuitable for human consumption. The waters of the *Alsietina* were used primarily for the irrigation of gardens and *naumachia*, mock naval battles conducted in artificial lakes (Frontinus 1899, 15).

In AD 52, the Emperor Claudius (10 BC to AD 54) completed the *Anio Novus* and the *Aqua Claudia* initiated by his predecessor, Caligula (AD 12 to 41). The waters of the *Aqua Claudi*a were derived from a spring and praised by Frontinus for their purity. The waters of the *Anio Novus*, like its predecessor, the *Anio Vetus*, were sourced from the Anio River. Despite the installation of a settling tank, water from the *Anio Novus* often reached Rome “in a discolored condition whenever there are heavy rains” (Frontinus 1899, 19). Construction of the *Aqua Traiana* began in AD 109 during the reign of Trajan (AD 53 to 117). The last of the 11 aqueducts of ancient Rome, the *Aqua Alexandrina* was built in AD 226.

It is not entirely clear that the average citizen of ancient Rome obtained most of their daily water supply from the aqueducts. Wells and cisterns were major sources of water (Niebuhr 1852, 390; Hodge 1992, 48; Wilson 2008). Certainly, the Romans were prodigious well diggers. At Saalburg, a Roman fort in Germany, excavations have found 99 wells (Hodge 1992, 57). A Roman well in Gaul reached a remarkable depth of 80 m (Wilson 2008, 286). Houses or apartment buildings in Rome usually had either a well or a cistern, and public wells were located throughout the city (Hodge 1992, 57).

Before the construction of the *Aqua Appia* in 312 BC, Frontinus informs us that “from the foundation of the city for 441 years, the Romans were content with the use of waters which they drew, either from the Tiber, or from wells, or from springs” (Frontinus 1899, 5). It seems likely that if the River Tiber ever supplied water to any significant extent, it must have been very early in Roman times. Like all surface water, the Tiber was surely contaminated by sewage. And as Rome is built on hills above the river, transporting water uphill surely would have been arduous. Most Romans probably obtained their daily water supplies from fountains supplied by aqueducts (Wilson 2008, 306). Frontinus enumerated 591 public water basins (*lacus*) in first century Rome (Frontinus 1899, 53). And the *insulae*, or apartment buildings, where most people lived, typically lacked cisterns (Scobie 1986, 424).

Water may not have even been the major beverage consumed by most Romans. It has been argued that the daily practice in the ancient world was to consume prodigious amounts of alcoholic beverages because uncontaminated water supplies were scarce (Vallee 1998). “Beer and wine were free of pathogens,” but wine was always diluted with water before consumption (Vallee 1998, 81). On the other hand, it is doubtful if much of the ancient Roman population could afford to purchase alcoholic beverages on a regular basis. Plutarch (c. 46 to 120 AD) informs us that when Cato the Censor was on military duty, “he usually drank water,” resorting to wine in small quantities only “if his strength was run down” (Plutarch 1906, 37).

**Figure 1:**
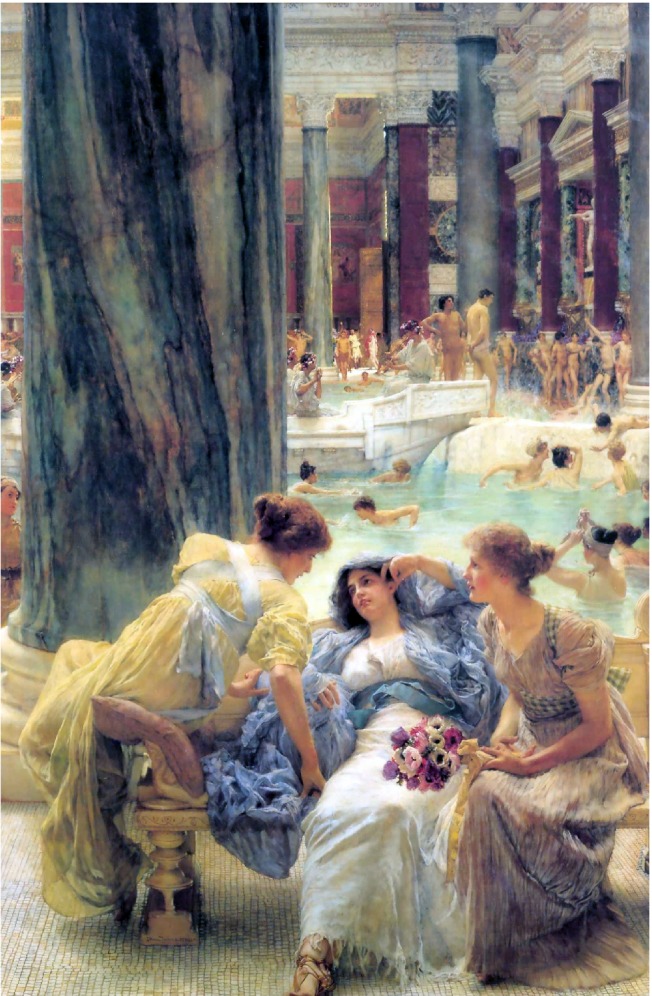
Baths of Caracalla, 1881 painting by Virgilio Mattoni de la Fuente (1842 to 1923), public domain.

Although the aqueducts were undoubtedly an important component of the daily household water supply in Rome, their most important function was to facilitate the Roman passion for bathing. It seems that the fascination with bathing was inherited from the Greeks. Public Greek bathing facilities date from the fifth century BC, and about 75 structures have been identified (Rogers 2018, 32). At least one contemporary scholar has concluded that bathing was “the greatest single reason” that aqueducts were built (Hodge 1992, 6). In 33 BC, there were 170 baths in Rome. At the height of the empire, the number approached 1000 (Carcopino 1940, 254). The grandest of the bathing facilities was the Baths of Caracalla (Figure 1), constructed early in the third century AD by the Emperor Caracalla (AD 188 to 217). To provide for the enormous amounts of water consumed by the Baths, Caracalla tapped an additional spring to supplement the *Aqua Marcia* aqueduct (Ashby 1935, 14). Large bath complexes could also be complemented by a reservoir cistern that was filled overnight so as to provide additional flow during daily operating hours (Wilson 2008, 305). Ancient Rome contained “a number of large cisterns and reservoirs … in which water could have been stored during the night” (Bruun 1991, 373).

The main building at Caracalla occupies an area of 2.4 ha and is surrounded by a complex of gardens and grounds with an area of 9 ha (Oetelaar 2014, 45). It has been estimated that Caracalla was able to accommodate as many as 10,000 people daily (Bruun 2013, 310). The Baths of Caracalla contained “every type of bath that ingenuity could devise” (Carcopino 1940, 256). These included a *natatio* (swimming pool), *caldarium* (hot room), *tepidarium* (warm room), and *frigidarim* (cold room). The hot baths were heated by means of a *hypocaust*, an under‐the‐floor central heating system. Lavish decorations included marble and mosaic floors, paintings, fountains, and sculptures (Delaine 1997, 24; Gensheimer 2018; Yegül 2010). Ancillary features of the bath complex included a library, rooms for exercise and massage, eateries, and a theater (Oetelaar 2014, 46; Carcopino 1940, 256). Neither was Caracalla unique in its opulence. “Excavations of baths all over the empire habitually turn up evidence of marble paneling, mosaics, painted stucco, and statuary” (Fagan 1999, 179). The Romans spared no expense or effort in the decoration of their bathing facilities. Upon visiting the villa of Scipio Africanus (236 to 183 BC), Seneca (c. 4 BC to AD 65) was shocked at the plainest and frugality of the bath. “Who is there in our time that would condescend to bathe in like manner? A man thinks himself poor and mean, unless the walls are decorated with large and precious embossments” (1786, 74).

It seems that in Republican days, men and women had separate bathing facilities. Writing circa 30 BC, Vitruvius noted that in the construction of baths “we must also see to it that the hot bath rooms in the women's and men's departments adjoin each other” (1960, 157). But during the first century AD, it became an accepted cultural practice for men and women to bath together fully nude (Fagan 1999, 24 to 28, Ward 1992, 134). Pliny the Elder (AD 23 to 79) noted that women bathed “in the company of men” (1857, 138) and the works of the poet Martial provide abundant evidence that this was routine and normal (Fagan 1999, 27). Ovid (43 BC to 17/18 AD) suggested that the baths often functioned as a rendezvous for lovers (1877, 458). The sexes were segregated again in the second century AD by order of Hadrian, emperor from AD 117 through 138. As the physical facilities could not have been completely rebuilt, this separation must have been achieved by designating distinct time periods for men and women to utilize the baths (Carcopino 1940, 258). It is unclear to what extent Hadrian's rule was followed.

Ancient Rome was far from an egalitarian society. Yet class distinctions apparently vanished when bathing. “Members of all socio‐economic levels, from emperor to beggar, congregated in the public baths where there was virtually no individual privacy” (Scobie 1986, 429). “Emperors and subjects bathed together” (Thomson 1859, 43). Larger baths were no doubt “noisy, vibrant places, with dinner parties meeting; bathers eating, drinking, and singing; vendors shouting; prostitutes strutting; and thieves prowling” (Fagan 1999, 38 to 39).

Most aqueducts were supplied by groundwater as opposed to surface water (Hodge 1992, 69). Before the ascent of the Romans, the Greeks evidently understood that groundwater flow could be provided by infiltration. Plato (428 to 348 BC) noted that “fountains and streams” resulted from rainwater being absorbed in valleys (1937, 523 [761]), and Aristotle (384 to 322 BC) recognized that “mountains and high ground, suspended over the country like a saturated sponge, make the water ooze out and trickle together in minute quantities but in many places” (1923, 349).

The most common source for an aqueduct was a spring (Hodge 1992, 72). And when the Romans tapped a spring for an aqueduct, they typically augmented the flow and supply by driving tunnels or *adits* into the surrounding terrain (Hodge 1992, 75). Aqueduct water was almost always hard, containing significant quantities of dissolved minerals.

Although today we associate Roman aqueducts with the remains of soaring arches and arcades, the most common form was a surface channel (Hodge 1992, 93). The channel was constructed of masonry, laid about 0.5 to 1.0 m below the ground, and was covered. Bottom and sides were lined with a waterproof cement. Aqueducts had to be large enough for human beings to enter and work. The *Aqua Marcia*, for example, was 0.9 m wide and 2.4 m in height (Hodge 1992, 94). Minimum aqueduct dimensions were determined not by the water flow, but by the need for human access and maintenance. The spring‐derived hard water flowing through most aqueducts deposited significant amounts of sinter over time—enough to reduce and choke off flow if not removed. The Roman aqueduct at Nîmes, France, accumulated a thickness of 0.46 m of sinter in about 200 years (Hodge 1992, 228). Frontinus informs us that “the maintenance of the works” was the most important part of his duties (Frontinus 1899, 19). Hundreds of slaves were employed on a regular basis to maintain and refurbish the aqueducts (Walker and Dart 2011, 9). During the reign of Claudius (41 to 54 AD), 460 people worked on the aqueducts. These included “overseers, reservoir‐keepers, line‐walkers, pavers, plasterers, and other workmen” (Frontinus 1899, 83). The expense of the workers as well as the cost of the materials was paid by the Emperor, but this was offset by revenues derived by selling water rights (Frontinus 1899, 85).

In the Republican period, aediles and censors seem to have been given the responsibility for constructing and maintaining the aqueducts and sewers. Appointed censor in 184 BC, Cato the Elder (234 to 149 BC), reportedly cut off aqueduct water “running or carried into any private building” (Livius 1823, 347). Presumably this action was only taken in the cases of people who were stealing water from the aqueducts. Theft of water by diversion was common and flagrant (Frontinus 1899, 51). When he assumed the post of water commissioner in 97 AD, Frontinus discovered that illegal diversions from the aqueducts were substantial, a problem he claimed to have solved. Theft could occur through unauthorized hookups in the city, or by diversions in the countryside. Frontinus reported finding “illicit pipes within the city” (Frontinus 1899, 43). He also found some farmers “whose fields border on the aqueducts, tap the conduits” (Frontinus 1899, 51). Legal water lines from the aqueducts to private properties could be obtained only by a grant from the Emperor. Presumably this favor was dispensed to curry political favor with powerful or wealthy individuals. The right to draw water directly from the public supply expired with the death of the grant holder. “The right to granted water does not pass either to the heirs, or to the buyer, or to any new occupant of the land” (Frontinus 1899, 77).

The inevitable accumulation of sinter had a benefit: it made the use of lead pipes (*fistulae*) practical and safe. Vitruvius acknowledged the poisonous properties of lead and argued that “water from clay pipes is much more wholesome than that which is conducted through lead pipes” (1960, 246). Yet the Romans made wide use of lead pipes. Lead was relatively inexpensive, malleable, flexible, and strong. If the water was hard, the interior of any lead pipe was soon insulated from contact with the water flowing through it by a layer of mineral deposits. To the extent that Romans may have accumulated excessive amounts of lead in their bodies, it is unlikely that the source was lead water pipes (Bruun 1991, 129). Pipes made of terracotta, stone, and wood were also used in Roman aqueducts and water supply. Wood was undoubtedly less durable than lead, but was often employed in smaller, isolated systems in the outer areas of the Roman Empire such as Germany (Hodge 1992, 111). Pliny the Elder noted that “the pine, the pitch‐tree, and alder are employed for making hollow pipes for the conveyance of water, and when buried in the earth will last for many years” (1892, 426).

**Figure 2:**
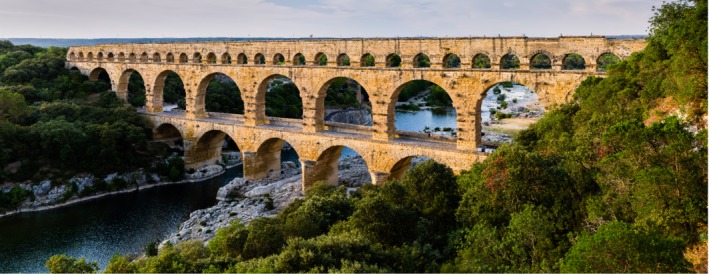
The Pont du Gard, a first‐century AD Roman bridge and aqueduct spanning the Gardon River near the town of Vers‐Pont‐du‐Gard in southern France. Photo by Benh Luei Song, licensed by CC BY‐SA 3.0, https://creativecommons.org/licenses/by-sa/3.0/deed.en.

All water flow was by gravity. If the gradient of the topography was not uniform, dips and hummocks had to be overcome by bridges, viaducts, tunnels, or siphons. Perhaps the most famous example of an aqueduct bridge is the Pont du Gard (Figure 2), an elegant structure that is a remarkable testament to the Roman ability to construct physical monuments that can withstand the ravages of time. Three tiers of arcades in the Pont du Gard reach a height of 49 m (Wilson 2008, 299). The Roman aqueduct at Lyon includes a siphon consisting of nine lead pipes laid side by side extending over a combined length of 16.6 km (Hodge 1992, 156). The typical Roman lead pipe was about 0.27 m in external diameter and strong enough to contain substantial water pressure. In general, the Romans used lead pipes everywhere in their hydraulic engineering in vast quantities (Hodge 1992, 15). The *Silvae* of Statius (c. AD 45 to 96) mentions a siphon pipe laid underneath the Anio River that supplied a villa owned by the patrician Manilius Vospiscus (1908, 61).

Upon arriving at Rome, aqueduct water typically flowed into a *castellum*, or settling tank (Rogers 2018, 25). From there, it was distributed through pipes (Wilson 2008, 302). Flow through the pipes was controlled by the diameter of an *ajutage* or *calix*, a bronze nozzle that connected lead pipes to a castellum (Hodge 1992, 295 to 296). Frontinus records that there were 25 standardized sizes of ajutages (Frontinus 1899, 33). Flow could be stopped or started with bronze stopcocks (Wilson 2008, 303). The Roman unit of area was the *quinaria*. One *quinaria* was a pipe 2.3125 cm in diameter (Hodge 1992, 299). Frontinus reports water discharges in units of quinaria (1899, 31). This is dimensionally incorrect, as water flow must have units of length cubed per unit time, and a *quinaria* has dimensions of length squared. The Romans had no means of measuring or metering flow velocities (Hodge 1992, 299). It seems that the Romans were not so much concerned with absolute volumetric discharges as relative discharges. A pipe with twice the area would carry twice the amount of water in a given time if the head gradients and other factors were equal.

Frontinus calculated the total discharge of all the aqueducts in Rome to be 14,018 *quinaria* (1899, 53). A modern estimate is that a pipe with a diameter of one *quinaria* will discharge 40 m^3^ in 24 h (Hodge 1992, 299; Bruun 1991, 385). This implies that the amount of water delivered daily to Rome near the end of the first century AD was 560,720 m^3^. Bruun (2013, 306 to 307) estimated a range of 520,000 to 635,000 m^3^ daily, while other scholars have estimated the daily supply to be as large as 1,000,000 m^3^ (Bruun 1991, 99). The population of Rome during the reign of Augustus (27 BC to AD 14) has been estimated to be in the neighborhood of 1 million inhabitants (Carcopino 1940, 18).

## Drains, Sewers, and Sanitation

**Figure 3:**
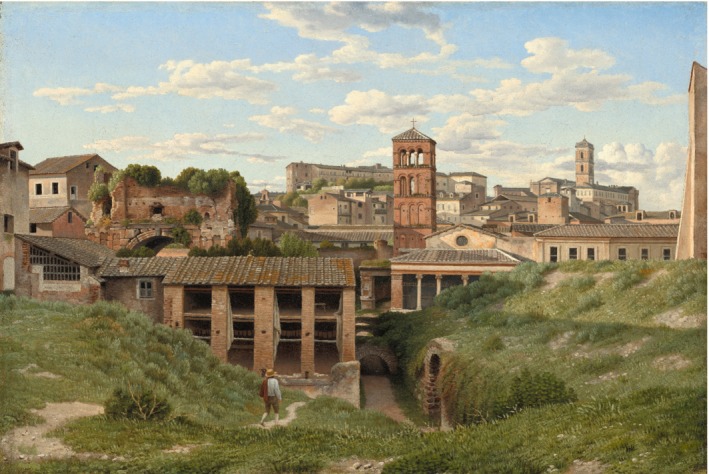
Outlet of the *Cloaca Maxima* sewer in Rome as it appeared in 1814. Painting by Christoffer Wilhelm Eckersberg (1783 to 1853), public domain.

The enormous flux of water entering Rome daily implies the existence of a corresponding system of drains and sewers to channel waste water and overflow to the Tiber. Indeed, the chief sewer in Rome, the *Cloaca Maxima*, preceded construction of the first aqueduct by several hundred years (Figure 3). The Romans did not invent the sewer. Effective sewer and drainage systems were constructed by the Minoan and Harappan civilizations in Crete and the Indus Valley as early as 3000 BC. But the Romans developed and improved earlier methods and enlarged the scale of such systems (De Feo et al. 2014).

Rarely has a sewer been the subject of literary adoration. Yet when Barthold Georg Niebuhr (1776 to 1831) described the *Cloaca Maxima*, he waxed poetic. “Even at the present day there stands unchanged the great sewer, the *Cloaca Maxima* … this work consisting of three semicircles of immense square blocks, which, though without mortar, have not to this day moved a knife's breadth from one another, drew the water from the surface, conducted it into the Tiber, and thus changed the lake into solid ground … [the] structure equals the pyramids in extent and massiveness, [and] far surpasses them in the difficulty of its execution” (1852, 52 to 53).

The *Cloaca Maxima* was originally constructed as a drainage channel, a “monumental, open‐air, fresh‐water canal” (Hopkins 2007, 1). The work was initiated before Rome became a Republic by the fifth king of Rome, Tarquinius Priscus, who reigned from 616 to 579 BC. According to Livy, Tarquinius constructed the sewer to drain water from “the lower parts of the city round the Forum, and the other valleys lying between the hills” (Livius 1896, 74). The work was difficult. Pliny noted that “Tarquinius Priscus … set the lower classes to work upon them, the laboriousness and prolonged duration of the employment became equally an object of dread to them; and the consequence was, that suicide was a thing of common occurrence” (1857, 347). Priscus' grandson, Tarquinius Superbus (reigned 535 to 509 BC), perfected “the works his grandfather had left half finished,” but the *Cloaca Maxima* was not covered until the second century BC (Dionysius 1758, 232). So for several hundred years the sewer was simply an open canal crossed by bridges (Hopkins 2007, 9).

Most people in ancient Rome lived in *insulae*, apartment houses, rather than *domus*, private homes. A fourth century AD accounting lists “one private house for every 26 blocks of apartment houses” (Carcopino 1940, 23). Even Senators often rented rooms in apartment buildings (Scobie 1986, 401). And virtually none of these apartment houses had water supply systems or connections to the sewers (Koloski‐Ostrow 2011, 53). The poet Martial (c. 38 to 104 AD) complained that his house lacked a water supply. “My dry house complains that it is not refreshed even by the slightest shower, although the Marican fount babbles close by” (Martial 1904, 401). If a facility lacked a well or cistern, occupants had to draw their water from the closest public fountain. Although connecting to the public sewers was not illegal, there were technical problems. Gas traps were unknown in Roman plumbing, and connection to a sewer would have exposed residential occupants to offensive odors and the risk of explosions from hydrogen sulfide and methane (Scobie 1986, 412).

Public toilets were commonly located near markets or baths and almost always connected to the city water system and sewers. Waste water from a bath or overflow from a fountain would have been ideal for flushing a public toilet (Jansen and Van Vaerenbergh 2011). Like the baths, toilets could be ornate. A *forica* near the Forum had marble toilet seats and “niches containing statues of gods and heroes” (Carcopino 1940, 41). But very few private dwellings were connected to the public sewers (Carcopino 1940, 40; Scobie 1986, 409). At Pompeii, human waste was usually disposed of by dumping it in cesspits, simple holes in the ground located in rooms about a meter square (Scobie 1986, 409). No latrines were flushed by water. When the cesspit reached a certain degree of fullness, the contents were evidently sold to manure merchants (*stercorarii*) who in turn peddled feces as agricultural fertilizer (Wilson 2011). There is little evidence for the existence of private latrines at Rome. The implication is that most people must have used the public facilities (Scobie 1986, 415). Chamber pots were probably also used, and may have been emptied into the streets. And “there is abundant evidence showing that many people relieved themselves in streets, doorways, tombs, and even behind statues” (Scobie 1986, 417).

The Romans were surely ignorant of the germ theory of disease, but knew empirically that water in the public baths was capable of inducing infection. In *De Medicina*, the physician Celsus (c. 25 BC to AD 50) warned that “bathing, before the wound is pure, is one of the very worst things that can be done: for it makes it humid and foul, and then gangrene is usually the consequence” (1831, 192). The sanitary conditions in Roman baths “left a lot to be desired” (Fagan 1999, 188). Public baths in Rome were contaminated with “a mixture of oil, sweat, and dirt” (Rogers 2018, 44). Marcus Aurelius (121 to 180 AD) referred to bath water as consisting of an “offensive mixture” of “oil and sweat, dirtiness and water” (Aurelius 1887, 128 to 129).

Frontinus attributed greater health and cleanliness in Rome to increased aqueduct flow (1899, 61). Overflow was both deliberate and necessary. Frontinus argued “there must necessarily be some overflow from the delivery tanks, this being proper not only for the health of the city, but also for use in the flushing of the sewers” (1899, 81). Although the aqueduct system supplied ancient Rome with abundant fresh and flowing water, sanitary conditions in ancient Rome were nevertheless severely lacking by modern standards. “The Roman world was indeed not as clean as modern audiences have come to believe” (Rogers 2018, 40). Toilets and sewers offered “a very high risk of contamination” (Jansen 2011, 162).

Ancient Romans evidently lacked a full comprehension of the dangers inherent in handling human waste. Human excrement contains organisms capable of causing “diarrhea, bacillary dysentery, infectious hepatitis, salmonella infection, and many other illnesses” (Scobie 1986, 407). Contact with fecal matter placed residents at high risk for a variety of infectious and deadly diseases, as well as parasitic infection (Scobie 1986, 421). Romans were subject to infection by whipworms, roundworms, the organism that causes dysentery, as well as fleas, head lice, body lice, pubic lice, and bed bugs (Mitchell 2016, 48). As a consequence, life expectancy at birth was no more than 20 or 30 years (Hopkins 1966, 264).

The modern concept of hygiene dates from the middle nineteenth century (Jansen 2011). From Roman times, sanitary conditions and life expectancy did not improve significantly until after the Industrial Revolution and the publication of Edwin Chadwick's (1800 to 1890) *Sanitary Report* in 1842. Although still lacking the term theory of disease, Chadwick connected “ravages of epidemic, endemic, and contagious diseases” with insufficient “drainage of houses, streets, roads, and land … [and] means of cleansing and removing solid refuse and impurities by available supplies of water” (1842, 4).

## Decay and Renaissance Revival

In 537 AD the Goths lay siege to Rome. According to Procopius (c. 500 to 565 AD), the Goths “tore open all the aqueducts, so that no water at all might enter the city from them” (Procopius 1919, 169). The damage was further compounded when the commanding Roman general, Belisarius, blocked the aqueducts “to prevent anyone from entering through them from the outside to do mischief” (Procopius 1919, 170). By the early seventh century, only the *Aqua Virgo* was functioning with any reliability. The other aqueducts, “patchily repaired after being frequently cut, and improperly maintained, leaked and formed marshes under their junctions” (Llewellyn 1970, 97). In a letter of AD 602, Pope Gregory I described the aqueducts as “so scorned and neglected that, unless greater attention be given to them, within a short time they will go utterly to ruin” (1898, 89).

Throughout the Middle Ages, Popes fought a losing battle to maintain and repair the aqueducts. In the early eighth century AD, Pope Gregory II restored the water supply to the baths at San Lorenzo fuori le Mura, a church in Rome (Coates‐Stephens 1998, 172). In the 770 s, Pope Hadrian I made major repairs to four aqueducts that had been out of commission for the previous 20 years (Coates‐Stephens 1998, 172). The implication is that by this time, only four aqueducts continued to function. In the ninth century, broken aqueducts “dripped and formed stagnant, fever‐carrying swamps” (Llewellyn 1970, 194). By the eleventh and twelfth centuries, references to functioning aqueducts had all but disappeared. By late medieval time, the only aqueduct functioning in Rome was the *Aqua Virgo* (Rinne 2010, 34).

**Figure 4:**
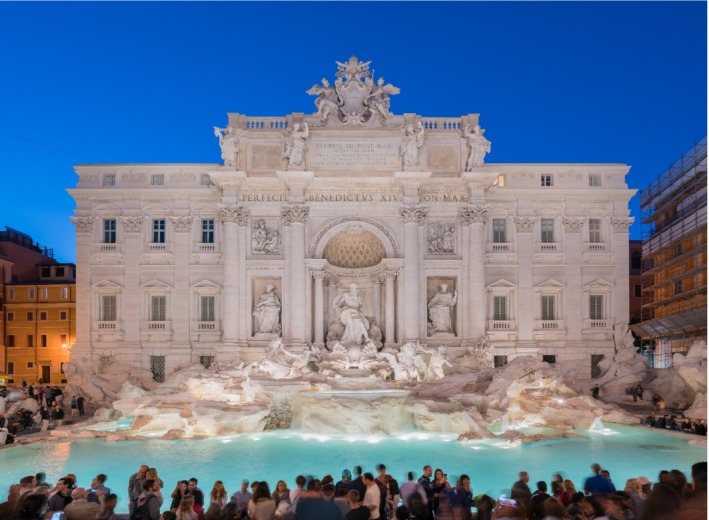
Trevi Fountain in Rome. Photo in 2015 by Livioandronico2013. Licensed by CC A‐SA 4.0, https://creativecommons.org/licenses/by-sa/4.0/deed.en.

In the fifteenth century, Pope Nicholas V (1397 to 1455) initiated the restoration of the *Aqua Virg*o. But the process of restoration and revival began in earnest in the late sixteenth century (Long 2018). By 1630, water flowed from more than 80 public fountains and “hundreds of private ones” (Rinne 2010, 4). The restoration of the *Aqua Virgo* was completed under the direction of Pope Pius V in 1570 (Martini 1976, 564). The aqueduct terminated in the predecessor of the Trevi Fountain. In 1732, Pope Clement XII sponsored a design contest for a new fountain that was won by Nicola Salvi (1697 to 1751). The Trevi Fountain (Figure 4) was completed in 1762, and it stands today as perhaps the most famous and beautiful fountain in the world (Rinne 2010, 226).

## Conclusion

Although today we tend to associate the aqueducts of ancient Rome with the Roman prowess in civil engineering and monumental construction, the fact that most aqueducts drew their water from springs is a testament to the importance of groundwater in sustaining human civilization. Groundwater remains a vital human resource today. As of 2015, the US Geological Survey estimated that 325 million people in the United States daily withdrew 321 billion liters of groundwater for public and domestic water supply, irrigation, watering of livestock, aquaculture, mining, industrial purposes, and thermoelectric power (Dieter et al. 2018, 7).
